# *Fasciola gigantica* vaccine construct: an in silico approach towards identification and design of a multi-epitope subunit vaccine using calcium binding EF-hand proteins

**DOI:** 10.1186/s12865-022-00535-y

**Published:** 2023-01-05

**Authors:** Kanhu Charan Das, Ruchishree Konhar, Devendra Kumar Biswal

**Affiliations:** 1 Bioinformatics Centre, North-Eastern Hill University, Shillong, Meghalaya India; 2 Informatics and Big Data, CSIR-Institute of Genomics and Integrative Biology, Delhi, India

**Keywords:** Vaccine, Calcium binding EF-hand protein, Fasciola gigantica, Fasciolosis, Immunoinformatics

## Abstract

Continuous attempts have been made to pinpoint candidate vaccine molecules and evaluate their effectiveness in order to commercialise such vaccines for the treatment of tropical fascioliasis in livestock. The pathophysiology of fascioliasis can be related to liver damage brought on by immature flukes that migrate and feed, as well as immunological reactions to chemicals produced by the parasites and alarm signals brought on by tissue damage. Future research should, in our opinion, concentrate on the biology of invasive parasites and the resulting immune responses, particularly in the early stages of infection. The goal of the current study was to use the calcium-binding proteins from *F. gigantica* to create a multi-epitope subunit vaccine. The adjuvant, B-cell epitopes, CTL epitopes, and HTL epitopes that make up the vaccine construct are all connected by certain linkers. The antigenicity, allergenicity, and physiochemical properties of the vaccine construct were examined. The vaccine construct was docked with toll-like receptor 2, and simulations of the molecular dynamics of the complex's stability, interaction, and dynamics were run. After performing in silico cloning and immunosimulation, it was discovered that the construct was suitable for further investigation. New vaccination technologies and adjuvant development are advancing our food safety procedures since vaccines are seen as safe and are accepted by the user community. This research is also applicable to the *F. hepatica* system.

**Supplementary Information:**

The online version contains supplementary material available at 10.1186/s12865-022-00535-y.

## Introduction

Fascioliasis is a *food*-borne disease caused by two *Fasciola* species namely *Fasciola hepatica* and *Fasciola gigantica*. *F. hepatica* is broadly found in America, Europe and Oceania while *F. gigantica* is reported in Africa, South Asia, South-East Asia, and Far East. It is categorized as a neglected tropical disease that affects the sheep, cattle and humans. Approximately 2.4 million people are infected worldwide in 70 countries as per World Health Organization (WHO) reports [1]. Human infections are increasingly reported in Europe, America, Africa and South East Asia [2]. Fascioliasis severely affects milk and meat production [2, 3]. Currently the drug Triclabendazole (TCZ) is administered against the fascioliasis. Looking into the increasing number of cases in animals as well as humans and resistance to TCZ in parasites, the only alternative way to control this food-borne disease is vaccination [4]. These parasites enter the host system through the peritoneal cavity to the liver, and finally to the bile ducts by manipulating cellular responses via immune evasion and subversion [5–9]. The calcium binding protein is very essential to the helminthic parasitic cells. The intracellular calcium ion concentration is closely regulated and it transiently increases in response to extracellular signals. The Ca^2+^ gives message to specific sub-cellular components by some calcium binding proteins, called Calmodulins, a ubiquitous intracellular calcium binding protein. Additionally, these proteins control specific calcium dependent processes, such as muscle contraction, mediated by troponin C and the light chain of myosin. In Schistosomes, the regulation of calcium levels is also important. Electron probe X-ray analysis of adult female and adult male worms reveal more calcium levels in females. This can be attributed to the calcareous corpuscles found in stage 4 vitelline cells of the female reproductive tract and thus involve a specific mechanism for calcium accumulation in these cells. Recent studies also establish high levels of calcium in the secretory bodies in the pre-acetabular glands of cercariae [10–12]. There are research studies on platyhelminthes which support Tegumental calcium-binding EF-hand protein to be an essential protein and a potential vaccine candidate against these parasites [13, 14]. Recently due to advances in computational methods there are several immunoinformatics tools that can predict antigenicity, allergenicity and epitopes with great precision. The present study deals with multi epitope prediction (B-cell, CTL and HTL epitopes) of vaccine design from three calcium binding proteins in these parasites.

## Materials and methods

### Sequence retrieval

The fasta formatted sequences in the present study were retrieved from National Centre for Biotechnology Information (NCBI) (AAZ20312.1, AEX92828.1, AEX92829.1). The structure of TLR2 (PDB ID: 5D3I) was downloaded from Protein Data Bank (PDB). The antigenicity of CABP was predicted on the online server (http://imed.med.ucm.es/Tools/antigenic.pl).

### Epitopes prediction

The ABCPred tools (https://webs.iiitd.edu.in/raghava/abcpred/ABC_submission.html) was used for the prediction of the B-cell epitopes for the three proteins that harnessed the B-cell epitope database (BCIPEP) and artificial neural network (ANN) [15]. This tool contains different epitopes from viruses, bacteria and parasites. The helper T-lymphocyte (HTL) epitopes were selected from MHC-II tools that used the immune epitope database (IEDB, http://tools.iedb.org/mhcii/). All the parameters were set to default except the allele LA-DPA1*01:03/DPB1*02:01 and HLA-DRB1*07:01, covering 99.9% of the world's population [16, 17]. The epitopes were selected on the criteria of lowest percentile rank and IC50. NetCTL1.2 tools was used to predict CTL epitopes (http://www.cbs.dtu.dk/ services/NetCTL/), a default thershold value 0.75 and three allele super types (A2, A3 and B7) having 88.3% population coverage [18] was set in the tools. The IFN-γ inducing epitope prediction was used to find the positive and negative inducer (http://crdd.osdd.net/raghava/ifnepitope/predict.php) [19]. The parameters were kept default except the Motif and SVM hybrid and IFN-γ versus other cytokine model.

### Toxicity of the selected epitopes

The selected epitopes were checked for toxicity by using ToxinPred module (http://crdd.osdd.net/raghava/toxinpred/multi_submit.php) to reveal the toxic or non-toxic nature of the individual epitopes [20].

### Vaccine design

The vaccine was designed using the adjuvant followed by B-cell, CTL and HTL epitopes joined by KK, AAY and GPGPG linkers respectively. Linkers were used to supple the amino acid into favorable conformations. An Adjuvant is used to boost the immunogenicity of the respective vaccine.

### Antigenicity, allergenicity and physiochemical parameters

Antigenicity is the capacity of binding the B-cell or T-cell receptors. ANTIGENpro and vaxijen (http://www.ddg-pharmfac.net/vaxijen/VaxiJen/VaxiJen.html) [21, 22] tools were used to find out the antigenicity of the vaccine. To elucidate the allergenicity of the vaccine construct, the Allertop (https://www.ddg-pharmfac.net/AllerTOP/) [23, 24] and Algpred (https://webs.iiitd.edu.in/raghava/algpred/submission.html) server was used [25]. Physiochemical parameters were harnessed by using the Expaxy Protparam Tools (https://web.expasy.org/protparam/) [26].

### Prediction of structure of the vaccine, refinement, validation and docking

To predict the tertiary structure of the construct vaccine, Raptor-X server (http://raptorx.uchicago.edu/StructurePrediction/predict/) was used [27]. Ramchandran plot was predicted using the Pdbsum [28] and ProsA was used to validate the structure [29]. Further the model was refined in Galaxy server [30] and the best model was chosen based on RMSD. TLR2 (PDBID:5D3I) was downloaded from PDB; docking was performed in cluspro (http://nrc.bu.edu/cluster). Of the total number of models that was generated from cluspro, the best model was chosen in binding energy to bind the vaccine construct [31, 32].

### Molecular dynamics simulation of the TLR2-vaccine complex

The molecular dynamics simulation (MDS) was performed on a 4 TF peak supercomputing power. The docked complex was kept in TIP3P water model and amber99SB force field. The genion tool was used to add 7 CL^−^ for neutralizing the system and for removing the steric clashes of the systems they were put in energy minimization and force below 1000 kj mol^−1^ nm^−1^. Particle Mesh Ewald (PME) method was used to calculate the long-range electrostatics, Lennard–Jones and coulomb were calculated by the distance 1.0 nm. The bond lengths was constrained by using LINCS algorithms; SHAKE algorithm was used to find out water bonds. After energy minimization the system was equilibrated at 1 ns of NVT (constant number of particles, volume and temperature) and NPT (constant number of particles, pressure and temperature) and the system was run for 150 ns. The g_rms, g_rmsf and g_hbonds were used to calculate the root mean square deviation(RMSD), root mean square fluctuation (RMSF) and number of hydrogen bonds respectively [33, 34]. MMPBSA was performed for last 10 ns in g_mmpbsa tool [35].

### In sillico cloning

For in silico cloning in bacterial expression system, we optimized the codon usage which was carried out by java codon adaption tool (http://www.jcat.de/) with E. coli K12 strain as the host of choice. The parameters “avoid Rho independent terminators”, “avoid prokaryotic ribosome binding sites” and “avoid cleavage sites of restriction enzymes” were selected. The optimal CAI and GC content is 0.8–1.0 and 30–70%. Then the optimized vaccine was reversed and at the N and C terminal XhoI and BamHI restriction sites were added. pET28a(+) vector was used to insert the sequence [36–38].

### Immune simulation for vaccine efficacy

C-ImmSim server19 was used in silico to carry out immune simulations to further assess the immunogenicity and immunological response of the multi-epitope vaccination. We used three doses spaced four weeks apart. In order to simulate 1,050 simulation steps, injections containing 1000 vaccine proteins were given four weeks apart at 1, 84, and 168 time-steps (each time-step is similar to 8 h in real life). The remaining parameters were kept default [39].

## Results

### sequence retrieval

All the three sequences of calcium binding protein from F. gigantica were retrieved from NCBI (Table1), the antigenicity of the calcium binding protein was checked based on their antigenic scores to enhance the immunogenicity of the protein TLR-2 agonist Lipoprotein LprA (P9WK55). All these sequences were considered for further analysis and vaccine design.

**Table 1:** Protein name and NCBI Accession of the protein

Protein name	NCBI Accession	Antigenicity score
CABP1	AAZ20312.1	1.0117
CABP3	AEX92828.1	1.0111
CABP4	AEX92829.1	1.0057

### B-cell, HTL,CTL, toxicity predictions and vaccine construct

For B-cell epitope predictions a total of 3 epitopes were selected from three proteins on the basis of highest score (Table 2). The sequence of the calcium binding protein was submitted to IEDB MHC-II for prediction of HTL epitope. Three epitopes were selected from three proteins based on the lowest percentile score and low IC50 value. The eligible binders exhibited lowest percentile value (Table 3). The NetCTL1.2 server was used to find out the CTL epitopes. A total of twelve supertypes for MHC class were considered of which A2, A3 and B7 supertypes were used for vaccine construct. The threshold was set at a default value of 0.75 and 9 epitopes with highest score were selected (Table 4). The final epitopes selected for vaccine construct are three, three and nine for B-cell, HTL and CTL respectively. We checked the IFN-γ production that showed all the epitopes to be positive (Additional file 1: Table S1). Toxicity prediction revealed all the epitopes to be non-toxic under study (Additional file 1: Table S2).

**Table 2:** Selected B-cell epitopes for the proteins sequences

Protein name	Epitope	Score
CABP1	ESLIDWFMELDKNNDE	0.95
CABP3	RTSIKPKITFTKGQQE	0.92
CABP4	RVEKMEREEVRAGRGR	0.90

**Table 3:** Selected HTL epitopes for the protein sequences and their IFN-γ inducing properties

Sl.no	Allelle	Epitope	Method	Percentile Rank	IFN-γ
1	HLA-DPA1*01:03/DPB1*02:01	SYWMRFSHEPFMSIQ	Consensus	0.47	Positive
2	HLA-DPA1*01:03/DPB1*02:01	MQFSHEPFLSIQFRY	Consensus	0.10	Positive
3	HLA-DPA1*01:03/DPB1*02:01	MKFSHEPFMSLQFKV	Consensus	0.30	Positive

**Table 4:** Selected CTL epitopes for protein sequences

Super type	Epitope	Score
A2 supertype	QMISLFLEL	1.3123
	SLIDWFMEL	1.5533
	KMIQLFLQL	1.2864
A3 supertype	ATATRTSIK	1.3363
	KSKGVSDSK	0.9302
	RTAEMRVEK	1.1854
B7 supertype	MPVERQEVV	1.4569
	RVEQKQRAL	1.4635
	KPEDMNLVV	1.0066

The multi-epitope vaccine was designed by Adjuvant followed by B-cell, CTL, and HTL epitopes. A TLR-2 agonist Lipoprotein LprA (P9WK55) was used as an adjuvant to increase the immunogenicity of the vaccine. All the selected epitopes from B-cell (3-mers), HTL (3-mers) and CTL (9-mers) were joined by specific linkers. EAAAK was used between adjuvant and epitopes.

### Antigenicity, allergenicity and physiochemical characterization of the proposed vaccine construct

The antigenicity of the predicted vaccine construct was 1.0219 and 0.6138 in antigenpro and vaxijen server respectively. The allergenicity of the construct vaccine was determined by allertop and algPred that revealed the vaccine to be non-allergenic. The allergenicity score in AlgPred tools was − 0.6532. ProtParam was used to evaluate physiochemical properties of the vaccine construct. The molecular weight of the 469 amino acid protein was 50.62KDA, the theoretical PI was 8.52, and the instability index of the construct vaccine was 26.83. The alphatic index 78.08 confirmed the thermostability of the protein. The GRAVY score of − 0.216 vouched for the hydrophilic nature of the protein. Thus the constructed vaccine is highly acidic, thermostable and hydrophilic in nature and is ideally suitable for further investigation for its efficacy.

### Vaccine modeling and validation

**Fig. 1:**
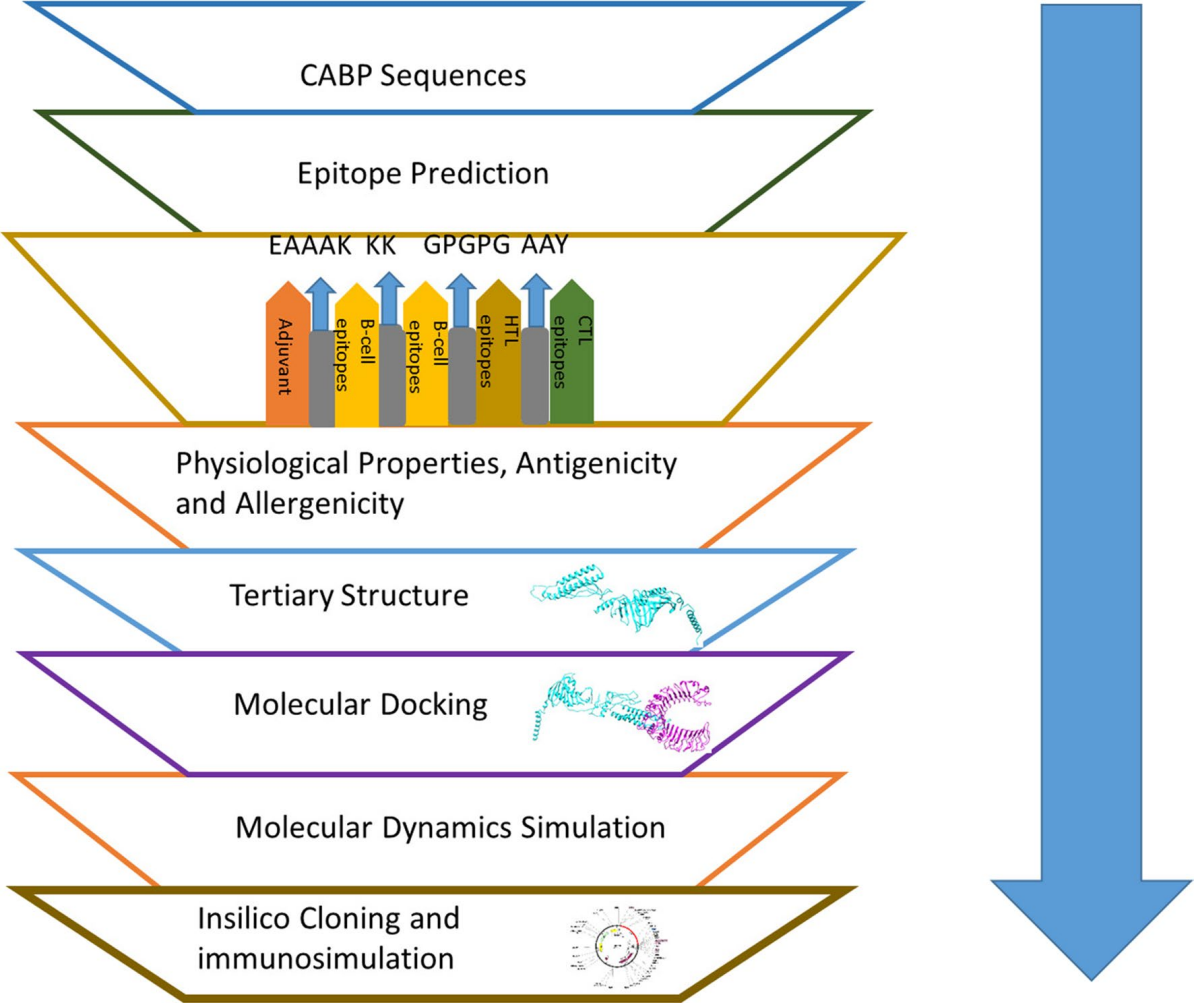
Schematic representation of the multi-epitope subunit vaccine candidate designing using B-cell, CTL and HTL epitopes followed by molecular docking, molecular dynamics simulation and in silico cloning

**Fig. 2.**
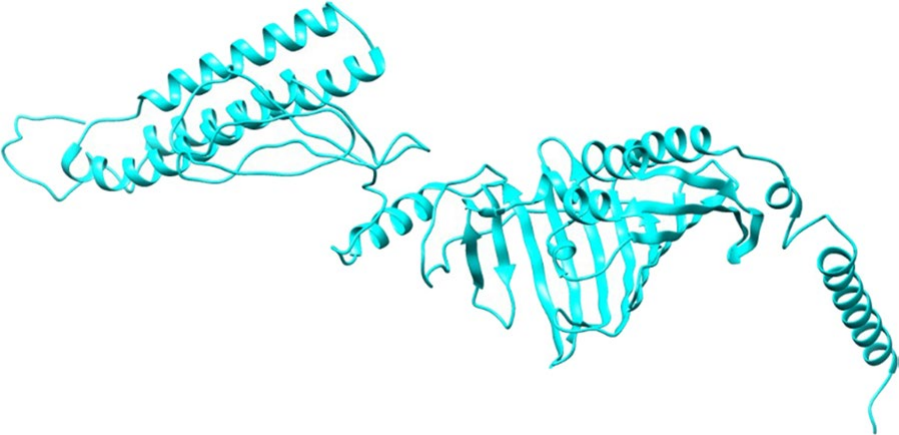
3-D structure of the Vaccine construct

The 3-D model of the multi-epitope vaccine construct was predicted by Raptor-X (Fig. 1) and further refined in Galaxy server to obtain a valid structure. The Ramachandran plot obtained from the said model was 87.6%, 11.2%, 0.7% and 0.5% in the most favored regions (Additional file 1: Fig. S1A), additionally allowed region, generously allowed region and disallowed region respectively (Fig. 2). After refinement the validated structure showed 90.8% in the most favored regions, 8.3% in additional allowed region, 0.5% in generously allowed region and 0.5% in disallowed region (Additional file 1: Fig. S1B)The prosa server revealed (Z-score = − 8.25) (Additional file 1: Fig. S2) and confirmed the qualitive nature of the vaccine construct. The energy plot vouched for the thermodynamically stable nature of the refined structure suitable for further docking analysis (Additional file 1: Fig. S3).

### Molecular docking with vaccine construct and TLR2

**Fig. 3:**
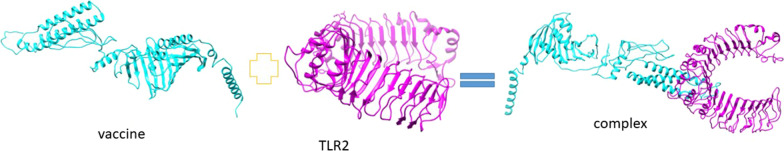
Molecular Docking of the TLR2 and vaccine complex

Molecular docking was performed to study the interaction between the TLR2(5D3I) and the vaccine construct. In total 16 models were obtained and the best model was selected based on the lowest binding energy (− 1209.2 kJ mol^−1^) for dynamics study between TLR2 and vaccine (Fig. 3).

### Dynamics study of the complex

**Fig. 4:**
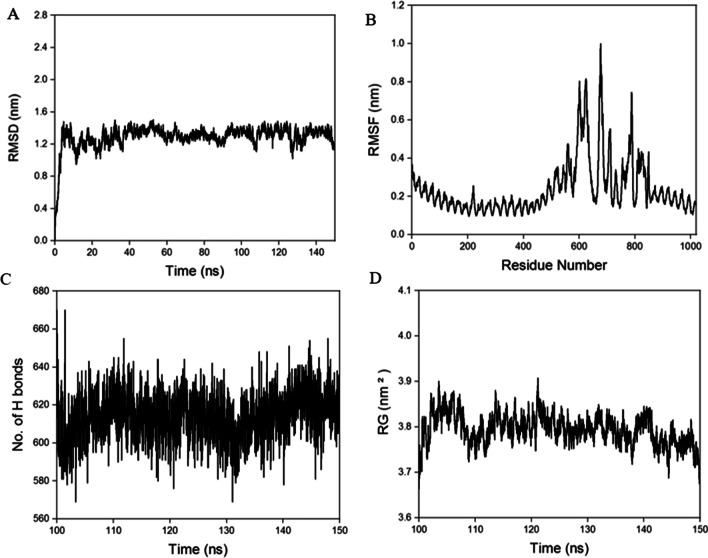
MD simulation of complex. **A** RMSD for the amino acid backbone of the complex, **B** RMSF of amino acids side chain of the same **C** Number of hydrogen bonds formed during last 50 ns, **D** Radius gyration (R_g_) of the complex

The molecular dynamics simulation (MDS) was performed for 150 ns for the TLR2-vaccine complex to understand the stability and thermodynamics of the complex. The RMSD (root mean square deviation), RMSF (root mean square fluctuation), Rg (radius of gyration), and the hydrogen bond was calculated. Cα atoms were analyzed for the RMSD. Figure 4A reveals that after 50 ns the trajectory was stable and average. RMSD was 1.29 nm. The RMSF gives a total idea about fluctuation of the residues and that with an average RMSF of 0.24 nm the system is confirmed to be stable (Fig. 4B) The hydrogen bonds gave an idea about the stability of the protein–protein complex with an average hydrogen bond of 616 (Fig. 4C) during the last 50 ns of the complex. The radius of gyration (Rg) values showed the compactness of the protein with an average Rg score of 3.79 for the complex (Fig. 4D). The MMPBSA data showed all the energy are in negative. The electrostatic, VDW and PB energy of vaccine and TLR2 complex is − 238.94, − 249.57 and − 25,654.18 respectively (Table5).

**Table 5:** Vanderwal, Electrostatic and PB energy of the complex from MMPBSA

MMPBSA	
Name	Energy in kj/mol
VDWAALS	− 249.5
EEL	− 238.9
PB Energy	− 25,654.1

### Immune simulation for vaccine efficacy

Significantly greater than the primary response were the secondary and tertiary ones. The presence of IgM + IgG, IgM, IgG1 + IgG2, and IgG1 antibodies was followed by a decrease in the antigen concentration (Additional file 1: Fig. 4). As a result, there was an increase in the population of active b-cells. Similar behavior between T helper (Th) and T cytotoxic cells was found (Additional file 1: Fig. 5). The fact that macrophage activity increased (Additional file 1: Fig. 6) emphasizes the vaccine's ability to induce long-lasting and efficient immunity.

### In silico cloning into pET28 (+) vector

**Fig. 5:**
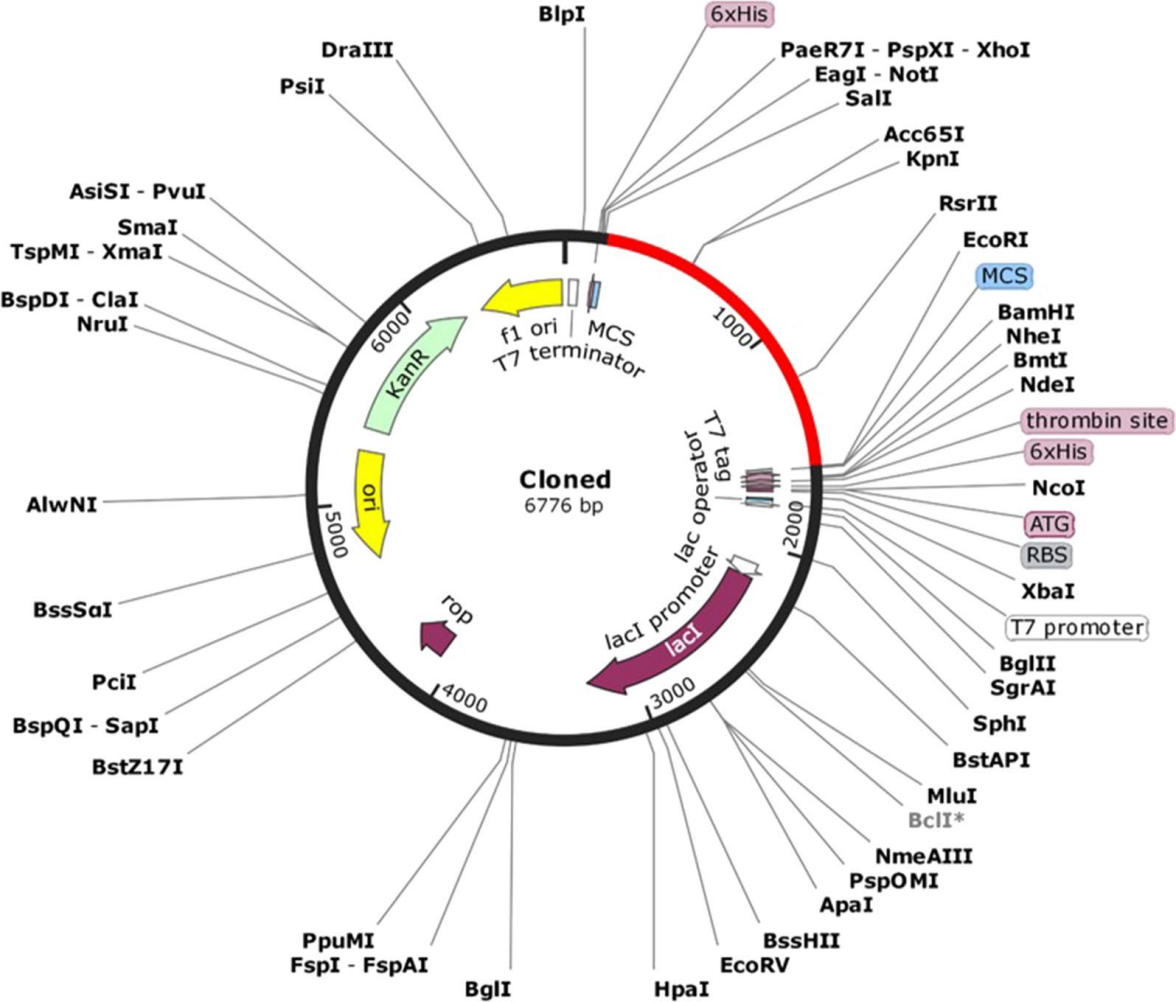
In silico cloning map showing the insert of vaccine protein specific optimized codons (red) into the pET28a (+) expression vector

pET28 (+) vector was used to clone the vaccine, with a Codon Adaption Index (CAI) of 1.0 and 50.73 GC content indicating its higher expression probability in *E. coli* cells. The restriction sites were absent in construct vaccine, so XhoI and BamHI restriction sites were added in N-terminal and C-terminal regions (Fig. 5) and the restriction clone with length 6776 base pairs was obtained by using the SnapGene1.1.3 restriction cloning tool.

## Discussion

Fasciolosis, often known as liver fluke disease, is a zoonotic condition brought on by the trematode *F. gigantica*. Fasciolosis is becoming more common due to the emergence of parasites resistant to the first-line medicine triclabendazole (TCBZ) and climate changes that are beneficial for the survival of the parasite's intermediate host. This rise in *F. gigantica* prevalence exacerbates the parasite's economic repercussions and shows that the liver fluke's crippling effects are not adequately represented.

Developing an efficient vaccine against *F. gigantica* has always remained a major challenge in the scientific arena for various reasons. High incidence of reinfection in endemic areas, anti-helminthic resistance, and acute infection cases resulting in animal death has posed greater challenges in production of novel vaccines against fasciolosis [40, 41]. Till date, the most important challenge in vaccine design against this trematode is the possibility to combat the Th2-type or immunosuppressive responses so that an efficient response to eliminate the parasite can be elucidated. There are several studies that attempted numerous vaccination regimes and have included different purified parasite molecules as well as recombinant varities in livestock with varying degrees of protection [42–45]. In a recent study, it was reported that a combination of two different vaccines each one composed by a cocktail of antigens (rCL1, rPrx, rHDM and rLAP) formulated in two different adjuvants (Montanide ISA 61 VG (G1) and Alhydrogel®(G2)) provides more protection in a vaccination trial performed in sheep [46]. As of now the vaccination trials developed against *F. gigantica* cannot be reproduced among animal models. A number of antigens have been tried as lead vaccine candidates in mice [47, 48].

Due to lack of improvement in animal survival, or hepatic damage most of these attempts have gone futile in the development of a commercially successful vaccine for livestock production. All these factors have necessitated defining new vaccine candidates and efficient adjuvant formulations using in silico strategies for the development of a novel vaccine design. Recently novel strategies have been adopted by developing advanced pipelines to address these issues. Wesołowska et al. [49] investigated the viability of cathepsins L (FhCL3-1, FhCL3-2) and B (FhCB3), which are released by juvenile liver flukes, as vaccine targets when given singly or in combination to rats. There is presently no licensed vaccination available to prevent *Fasciola hepatica* infections. However, there is an urgent need for a secure and complete fasciolosis vaccine given the alarming rise in medication resistance. It was proposed that focusing on fluke juvenile stages may be advantageous since control over the early immune response is essential for the parasite's establishment in its host. Additionally, it was predicted that particular antigens would work in concert to hinder liver fluke migration and hence lessen *F. hepatica* infection. Although the exact mechanism of protection is yet unknown, it appears to be dependent on cathepsin-specific antibodies produced by vaccination. The acquired results suggest that juvenile-specific cathepsins from *F. hepatica* are viable vaccine candidates because they successfully elicit immune responses that target the early migratory liver fluke stages [49].

Dogs are susceptible to the lethal canine circovirus (CanineCV), which affects both domestic and wild carnivores. There is neither a commercially available nor a clinically tested canine CV vaccination. To address this challenge, Kaushik et al., 2022, developed a multiepitope vaccine (MEV) construct targeting several CanineCV strains. Each strain of CanineCV's capsid and replicase protein were used to predict a total of 545 MHCII-binding CD^4+^ T cell epitope peptides. The final vaccine was created in silico employing a variety of antigenic, nontoxic, and conserved epitopes. Furthermore, reliable interactions between the projected MEV and the canine receptor TLR-5 were predicted using molecular docking and molecular dynamics simulations. One of the mapped epitope peptides was synthesized to confirm antigenicity and immunogenicity. The chosen epitope's in vivo investigation exhibited CD^4+^ T-cell-dependent antibody production, thereby confirming the MEV construct as a potential CanineCV vaccine candidate [50].

Orthohantavirus, a zoonotic virus that causes cardio-pulmonary illness in humans, is known to be a deadly condition. There is a continuing need to develop in-silico methods from the immunology domain to create the best possible peptide-based vaccine against it because there are few regimens to treat the illness and effective management to eradicate this dangerous virus. The nine-residue-long sequence "MIGLLSSRI," predicted and confirmed by the scientists, has the best interactions with HLA alleles of MHC Class II proteins, including HLA DRB1 0101, DRB1 0401, DRB1 0405, DRB1 0701, DRB1 0901, DRB1 1302, and DRB1 1501. According to molecular docking and simulation studies, this epitope possesses a selectable range of RMSD and RMSF values, satisfactory binding scores, an ACE value, and global energies for docked complexes. Joshi et al. [51] developed in silico studies using numerous tools, including AllergenFP, NETMHCII 3.2, VaxiJen, ToxinPred, PEPFOLD 3.5, DINC, IEDB-Population coverage, MHCPred, and JCat server, in their extensive study by harnessing statistical algorithms like HMM, ANN, ML, etc., that helped generate more accurate predictions of potential candidates for vaccine development. This novel methodology is simple, affordable, and quick, which will aid computational biologists to develop a vaccine to ward off this virus [51].

In Homo sapiens, the *Tropheryma whipplei* causes severe gastroenteritis and brain injury. The lower codon usage bias in *T. whipplei* as well as the 23S and 16S ribosomal RNA genes are indicated by the larger effective number of codon usage values. Genomic sets from *T. whipplei* were shown to have a minimal codon bias. Joshi et al. [51] studied the codon usage data using rare, extremely rare, and synonymous codons for predicting disease evolution, drug development and screening of vaccine candidates. The improvement of disease evolution prediction, prevention, and treatment would benefit from studies on genomics and codon adaptability. In addition to having a high codon usage frequency, amino acids like valine, aspartate, leucine, and phenylalanine were also discovered to be present in the epitopes KPSYLSALSAHLNDK and FKSFNYNVAIGVRQP that were screened from the proteins excinuclease ABC subunit UvrC and 3-oxoacyl-ACP reductase FabG, respectively. This approach brings up fresh possibilities for developing epitope-based peptide vaccines for many pathogenic species [52].

The rationale for the present vaccine design in this work was based on the properties of calcium binding EF-hand proteins. *Fasciola gigantica* secretes excretory-secretory proteins during infection to mediate its interaction with the host. Recently scientists have investigated immunomodulatory effects of a recombinant tegumental calcium-binding EF-hand protein 4 of *F. gigantica* (rFg-CaBP4) on goat monocytes. They carried out western blot analysis experiments in which the rat sera containing anti-rFg-CaBP4 polyclonal antibodies reacted with the isopropyl-ß-D-thiogalactopyranoside-induced rFg-CaBP4 protein. These findings substantiated the claims that calcium-binding EF-hand proteins play important roles in host-parasite interaction. To enhance our understanding of the strategies used by *F. gigantica* in evading host immune responses, characterization of the immunomodulatory role of rFg-CaBP4 should be undertaken [53].

The EF-hand motif is explicitly defined as a general and highly dynamic moiety in calcium-binding proteins. In the past few years the structures of these several complexes with target peptides and other compounds, have given enough insight into their recognition and interaction. X-ray structures of several of these proteins have been solved to high resolution, allowing an accurate description of the metal-binding sites, which would facilitate further computational work on the coordination of calcium and other metals. In Schistosomes possible functions of these tegument proteins are already investigated with in silico analyses including protein–protein functional interaction, site-specific variation and glycosylation modification. The analysis results have suggested that these tegument antigen proteins help Schistosomes in evading and modulating host immune responses for self-protection in the process of host-parasite interaction [54]

In the light of the above studies, we aimed to design a peptide-based vaccine against *F. gigantica* using immuno-bioinformatic tools. We identified B-cell, CTL and HTL epitopes from three calcium binding EF proteins from *F. gigantica* as discussed in the methodology section. All these epitopes were used to make a vaccine construct along with linkers by adding an adjuvant at the N-terminal to enhance its immunogenicity. We had validated and verified the quality of this vaccine construct using various bioinformatics tools and found that it is nontoxic, nonallergic, and thermally stable. Molecular docking was carried out, which confirmed its binding with a toll-like receptor-2 (TLR-2). The study of the proposed vaccine construct’s physiochemical characteristics has revealed that it is acidic, hydrophillic and highly stable as discussed elsewhere. The determined aliphatic index and scores for the instability index suggested that the protein of the vaccine could be stable and thermostable. The negative GRAVY score indicates its hydrophillic nature. It was found to be immunogenic, non-allergenic, and highly antigenic. This suggests the epitopic vaccine’s ability to produce a robust immune response with no allergic reactions.

The 3D structure prediction offered a detailed understanding of the spatial arrangement of essential protein components that served as an excellent base for the study of protein functions, protein components, ligands interactions and dynamics. Post refinement, the desirable features of the vaccine construct have improved reasonably. Ramachandran Plot analysis vouched for the majority of residues present in favoured and permitted areas, with very few residues in the disregarded sector, revealing the model to be of overall good quality. The appropriateness of these findings are further reinforced by RMSD value, RMSF, RG and Hydrogen bonds.

Thus the final formed structure was docked against TLR2 to test adequate binding to immediate immune response. Effective vaccine delivery into the body depends on the strong binding affinity of vaccine construct-TLR2 complex. The stability of vaccine-TLR-2 complex and underlying interactions were evaluated using molecular dynamic simulation. RMSD, RMSF, Hbond and RG data shows very good binding between the structure and TLR2. The MMPBSA data and immunosimulation shows that structure TLR2 complex is binding tightly and effectiveness for further study. The resulting CAI value was 0.1 and the GC content was also within an acceptable limit of 50.73%, implying possible higher expression in the *E*.*coli* K-12 system. An in silico cloning was carried out to check the expression of the final designed vaccine construct whose efficacy needs to be validated through wet bench experimental approaches.

Utilizing CABP vaccinations to protect against tropical fascioliasis infection was the goal of our study. We performed efficient antigenic receptor docking with the vaccine design to generate a CABP-TLR2 complex, which considerably increased the precision and breadth of our study. Therefore, a thoughtfully constructed in silico vaccine construct can save time and serve as a crucial tool against neglected food-borne tropical diseases. This study utilized a number of bioinformatics techniques and supports additional experimental validation.

## Supplementary Information

**Additional file 1: Table S1.** IFN-γ Results for the selected B-cell, HTL and CTL epitopes of CABP proteins. **Table S2.** ToxinPred results for the selected B-cell, HTL and CTL epitopes of CABP proteins. **Fig. S1.** (A) Ramachandran plot showing the presence of amino acid residues in favoured, allowed and outlier region, (B) after refinement Ramachandran plot showing the presence of amino acid residues in favoured, allowed and outlier region. **Fig. S2.** ProSA predicted 3D structure showed Z-score-8.25. **Fig. S3.** The energy plot for all residues showed that most of the residues lie in the negative region. Vaccine Sequence Adjuvant Followed by Bcell epitopes, HTL epitopes and CTL epitopes and the linker. **Fig. S4.** (A) Antigen and immunoglobulins, (B) production of cytokine and interleukins. **Fig. S5.** (A) B cell, (B) PLB cell population, (C) B cell population per state, (D) TH cell population, (E) TH cell population per state. **Fig. S6.** (A) TC cell population, (B) TC population per state, (C) NK cell population, (D) MA population per state, (E) DC population per state, (F) EP population per state.

## Data Availability

Antigenicity: (http://imed.med.ucm.es/Tools/antigenic.pl). ABCPred tools: (https://webs.iiitd.edu.in/raghava/abcpred/ABC_submission.html). IFN-γ Inducer: (http://crdd.osdd.net/raghava/ifnepitope/predict.php). ToxinPred: (http://crdd.osdd.net/raghava/toxinpred/multi_submit.php). Vaxijen: (http://www.ddg-pharmfac.net/vaxijen/VaxiJen/VaxiJen.html). Allertop: (https://www.ddg-pharmfac.net/AllerTOP/). Expaxy Protparam Tools: (https://web.expasy.org/protparam/). Raptor-X server: (http://raptorx.uchicago.edu/StructurePrediction/predict/). Java codon adaption tool: (http://www.jcat.de/). C-IMMSIM (https://kraken.iac.rm.cnr.it/C-IMMSIM/index.php?page=1).
