# Supplementary material for: Fasciola gigantica vaccine construct: an in silico approach towards identification and design of a multi-epitope subunit vaccine using calcium binding EF-hand proteins
Source: BMC Immunol. 2023 Jan 5;24:1. doi: 10.1186/s12865-022-00535-y (PMC9813462; doi:10.1186/s12865-022-00535-y)

*Fasciola gigantica* vaccine construct: an in silico approach towards identification and design of a multi-epitope subunit vaccine using calcium binding EF-hand proteins

Kanhu Charan Das^1^, Ruchishree Konhar^1,2^, Devendra Kumar Biswal^1^*

^1^Bioinformatics Centre, North-Eastern Hill University, Shillong, Meghalaya, India

^2^Informatics and Big Data, CSIR-Institute of Genomics and Integrative Biology, Delhi, India

***Correspondence:**Corresponding Author
[devbioinfo@gmail.com](mailto:devbioinfo@gmail.com); devendra@nehu.ac.in

Keywords: Vaccine, Calcium binding EF-hand protein, Fasciola gigantica, Fasciolosis, Immunoinformatics

**Supplementary Table 1.** IFN-γ Results for the selected B-cell, HTL and CTL epitopes of CABP proteins.

| **Serial No.** | **Epitope Name** | **Sequence** | **Result** | **Score** |
| --- | --- | --- | --- | --- |
| 1 | Seq1 | [RTSIKPKITFTKGQQE](https://webs.iiitd.edu.in/raghava/ifnepitope/pep_design.php?sequence=RTSIKPKITFTKGQQE&method=hybrid&model=rest) | POSITIVE | 0.58172937 |
| 2 | seq2 | [ESLIDWFMELDKNNDE](https://webs.iiitd.edu.in/raghava/ifnepitope/pep_design.php?sequence=ESLIDWFMELDKNNDE&method=hybrid&model=rest) | POSITIVE | 0.33406654 |
| 3 | seq3 | [RVEKMEREEVRAGRGR](https://webs.iiitd.edu.in/raghava/ifnepitope/pep_design.php?sequence=RVEKMEREEVRAGRGR&method=hybrid&model=rest) | POSITIVE | 0.49752939 |
| 4 | seq4 | [SYWMRFSHEPFMSIQ](https://webs.iiitd.edu.in/raghava/ifnepitope/pep_design.php?sequence=SYWMRFSHEPFMSIQ&method=hybrid&model=rest) | POSITIVE | 0.47819936 |
| 5 | seq5 | [MQFSHEPFLSIQFRY](https://webs.iiitd.edu.in/raghava/ifnepitope/pep_design.php?sequence=MQFSHEPFLSIQFRY&method=hybrid&model=rest) | POSITIVE | 0.49592012 |
| 6 | seq6 | [MKFSHEPFMSLQFKV](https://webs.iiitd.edu.in/raghava/ifnepitope/pep_design.php?sequence=MKFSHEPFMSLQFKV&method=hybrid&model=rest) | POSITIVE | 0.52790619 |
| 7 | seq7 | [QMISLFLEL](https://webs.iiitd.edu.in/raghava/ifnepitope/pep_design.php?sequence=QMISLFLEL&method=hybrid&model=rest) | POSITIVE | 0.473103 |
| 8 | seq8 | [ATATRTSIK](https://webs.iiitd.edu.in/raghava/ifnepitope/pep_design.php?sequence=ATATRTSIK&method=hybrid&model=rest) | POSITIVE | 0.45021284 |
| 9 | seq9 | [MPVERQEVV](https://webs.iiitd.edu.in/raghava/ifnepitope/pep_design.php?sequence=MPVERQEVV&method=hybrid&model=rest) | POSITIVE | 0.45920672 |
| 10 | seq10 | [SLIDWFMEL](https://webs.iiitd.edu.in/raghava/ifnepitope/pep_design.php?sequence=SLIDWFMEL&method=hybrid&model=rest) | POSITIVE | 0.4558736 |
| 11 | seq11 | [KSKGVSDSK](https://webs.iiitd.edu.in/raghava/ifnepitope/pep_design.php?sequence=KSKGVSDSK&method=hybrid&model=rest) | POSITIVE | 0.45059875 |
| 12 | seq12 | [RVEQKQRAL](https://webs.iiitd.edu.in/raghava/ifnepitope/pep_design.php?sequence=RVEQKQRAL&method=hybrid&model=rest) | POSITIVE | 0.4339003 |
| 13 | seq13 | [KMIQLFLQL](https://webs.iiitd.edu.in/raghava/ifnepitope/pep_design.php?sequence=KMIQLFLQL&method=hybrid&model=rest) | POSITIVE | 0.45926326 |
| 14 | seq14 | [RTAEMRVEK](https://webs.iiitd.edu.in/raghava/ifnepitope/pep_design.php?sequence=RTAEMRVEK&method=hybrid&model=rest) | POSITIVE | 0.46709254 |
| 15 | seq15 | [KPEDMNLVV](https://webs.iiitd.edu.in/raghava/ifnepitope/pep_design.php?sequence=KPEDMNLVV&method=hybrid&model=rest) | POSITIVE | 0.45572336 |

**Supplementary Table 2.** ToxinPred results for the selected B-cell, HTL and CTL epitopes of CABP proteins.

| [Peptide ID](http://crdd.osdd.net/raghava/toxinpred/multi_submitfreq_S.php?ran=39366) | [Peptide Sequence](http://crdd.osdd.net/raghava/toxinpred/multi_submitfreq_S.php?ran=39366) | [SVM Score](http://crdd.osdd.net/raghava/toxinpred/multi_submitfreq_S.php?ran=39366) | [Prediction](http://crdd.osdd.net/raghava/toxinpred/multi_submitfreq_S.php?ran=39366) |
| --- | --- | --- | --- |
| Seq1 | [RTSIKPKITFTKGQQE](http://crdd.osdd.net/raghava/toxinpred/pepsearch_S.php?seq=RTSIKPKITFTKGQQE&thval=) | -1.75 | Non-Toxin |
| seq2 | [ESLIDWFMELDKNNDE](http://crdd.osdd.net/raghava/toxinpred/pepsearch_S.php?seq=ESLIDWFMELDKNNDE&thval=) | -0.90 | Non-Toxin |
| seq3 | [RVEKMEREEVRAGRGR](http://crdd.osdd.net/raghava/toxinpred/pepsearch_S.php?seq=RVEKMEREEVRAGRGR&thval=) | -0.62 | Non-Toxin |
| seq4 | [SYWMRFSHEPFMSIQ](http://crdd.osdd.net/raghava/toxinpred/pepsearch_S.php?seq=SYWMRFSHEPFMSIQ&thval=) | -2.03 | Non-Toxin |
| seq5 | [MQFSHEPFLSIQFRY](http://crdd.osdd.net/raghava/toxinpred/pepsearch_S.php?seq=MQFSHEPFLSIQFRY&thval=) | -1.34 | Non-Toxin |
| seq6 | [MKFSHEPFMSLQFKV](http://crdd.osdd.net/raghava/toxinpred/pepsearch_S.php?seq=MKFSHEPFMSLQFKV&thval=) | -0.90 | Non-Toxin |
| seq7 | [QMISLFLEL](http://crdd.osdd.net/raghava/toxinpred/pepsearch_S.php?seq=QMISLFLEL&thval=) | -1.01 | Non-Toxin |
| seq8 | [ATATRTSIK](http://crdd.osdd.net/raghava/toxinpred/pepsearch_S.php?seq=ATATRTSIK&thval=) | -0.78 | Non-Toxin |
| seq9 | [MPVERQEVV](http://crdd.osdd.net/raghava/toxinpred/pepsearch_S.php?seq=MPVERQEVV&thval=) | -1.65 | Non-Toxin |
| seq10 | [SLIDWFMEL](http://crdd.osdd.net/raghava/toxinpred/pepsearch_S.php?seq=SLIDWFMEL&thval=) | -0.89 | Non-Toxin |
| seq11 | [KSKGVSDSK](http://crdd.osdd.net/raghava/toxinpred/pepsearch_S.php?seq=KSKGVSDSK&thval=) | -1.02 | Non-Toxin |
| seq12 | [RVEQKQRAL](http://crdd.osdd.net/raghava/toxinpred/pepsearch_S.php?seq=RVEQKQRAL&thval=) | -0.84 | Non-Toxin |
| seq13 | [KMIQLFLQL](http://crdd.osdd.net/raghava/toxinpred/pepsearch_S.php?seq=KMIQLFLQL&thval=) | -0.89 | Non-Toxin |
| seq14 | [RTAEMRVEK](http://crdd.osdd.net/raghava/toxinpred/pepsearch_S.php?seq=RTAEMRVEK&thval=) | -1.28 | Non-Toxin |
| seq15 | [KPEDMNLVV](http://crdd.osdd.net/raghava/toxinpred/pepsearch_S.php?seq=KPEDMNLVV&thval=) | -0.82 | Non-Toxin |

Supplementary Figure 1. (A) Ramachandran plot showing the presence of amino acid residues in favored, allowed and outlier region (B) after refinement Ramachandran plot showing the presence of amino acid residues in favored, allowed and outlier region.


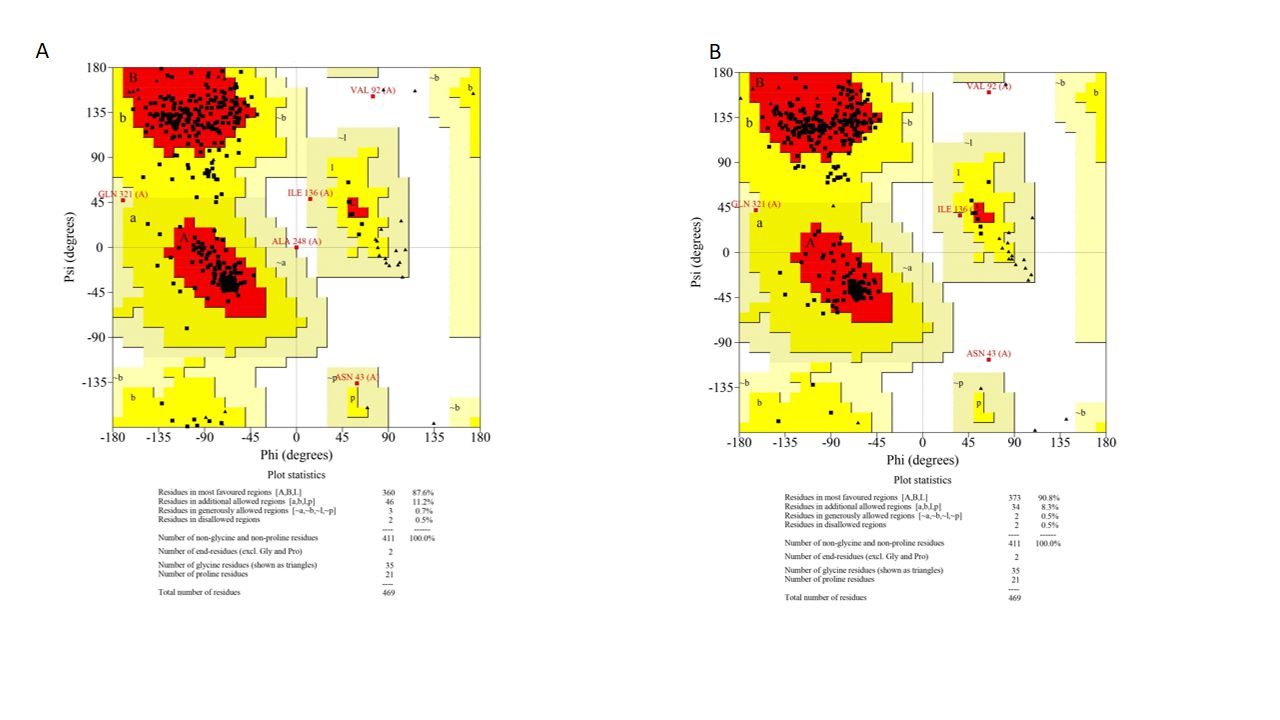


Supplementary Figure 2. ProSA predicted 3D structure showed Z-score -8.25


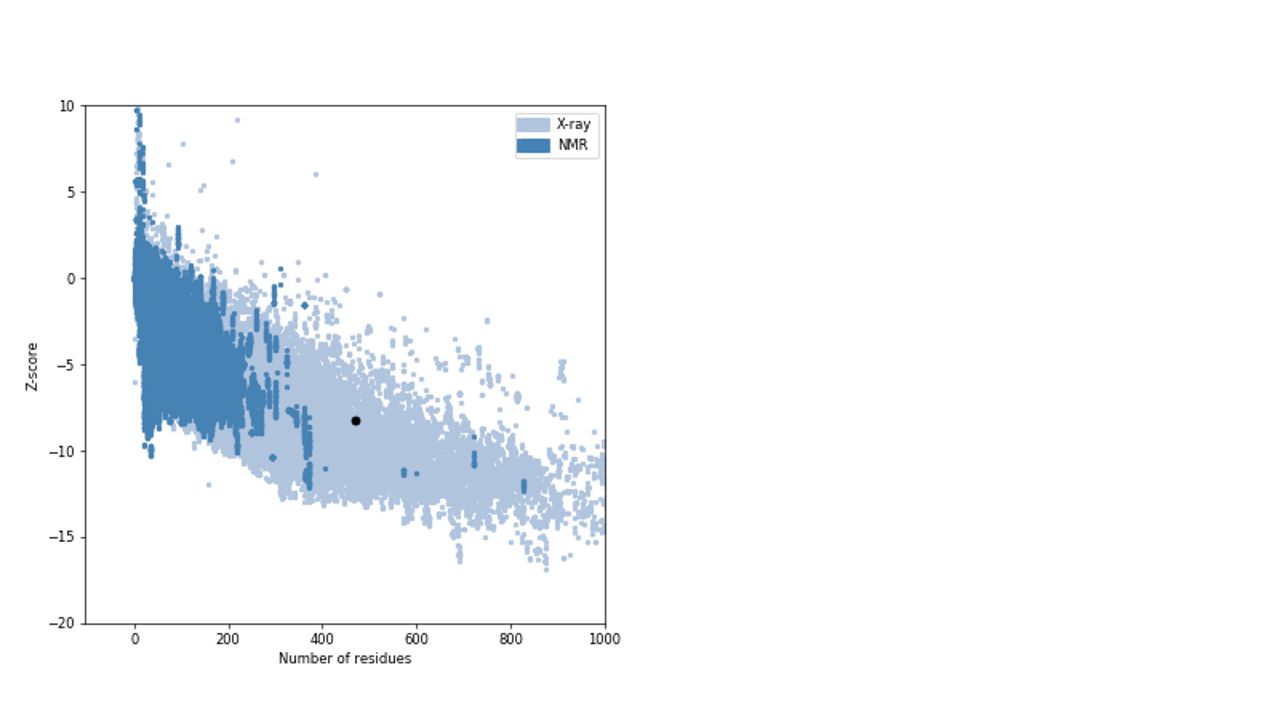


Supplementary Figure 3. The energy plot for all residues showed that most of the residues lie in the negative region.


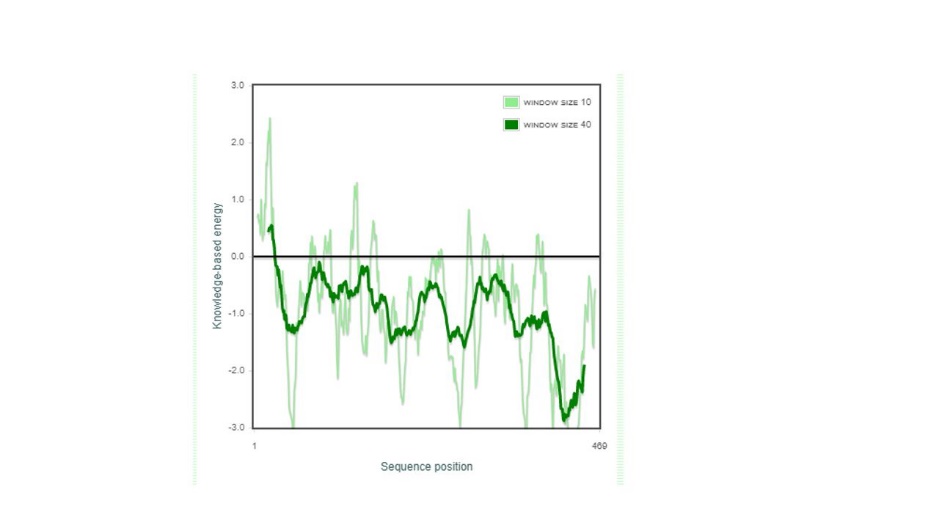


**Vaccine Sequence**

MKHPPCSVVAAATAILAVVLAIGGCSTEGDAGKASDTAATASNGDAAMLLKQATDAMRKVTGMHVRLAVTGDVPNLRVTKLEGDISNTPQTVATGSATLLVGNKSEDAKFVYVDGHLYSDLGQPGTYTDFGNGASIYNVSVLLDPNKGLANLLANLKDASVAGSQQADGVATTKITGNSSADDIATLAGSRLTSEDVKTVPTTVWIASDGSSHLVQIQIAPTKDTSVTLTMSDWGKQVTATKPVEAAAKRTSIKPKITFTKGQQEKKESLIDWFMELDKNNDEKKRVEKMEREEVRAGRGRGPGPGSYWMRFSHEPFMSIQGPGPGMQFSHEPFLSIQFRYGPGPGMKFSHEPFMSLQFKVAAYQMISLFLELAAYATATRTSIKAAYMPVERQEVVAAYSLIDWFMELAAYKSKGVSDSKAAYRVEQKQRALAAYKMIQLFLQLAAYRTAEMRVEKAAYKPEDMNLVV

Bright green colours is the adjuvant, Turquoise colours represents the Bcell epitope, Dark blue represents the HTL epitope,pink colours represents CTL epitopes and Yellow colours represents the linker

Supplementary Fig.4(A)Antigen and immunoglobulins (B)production of cytokine and interleukins.


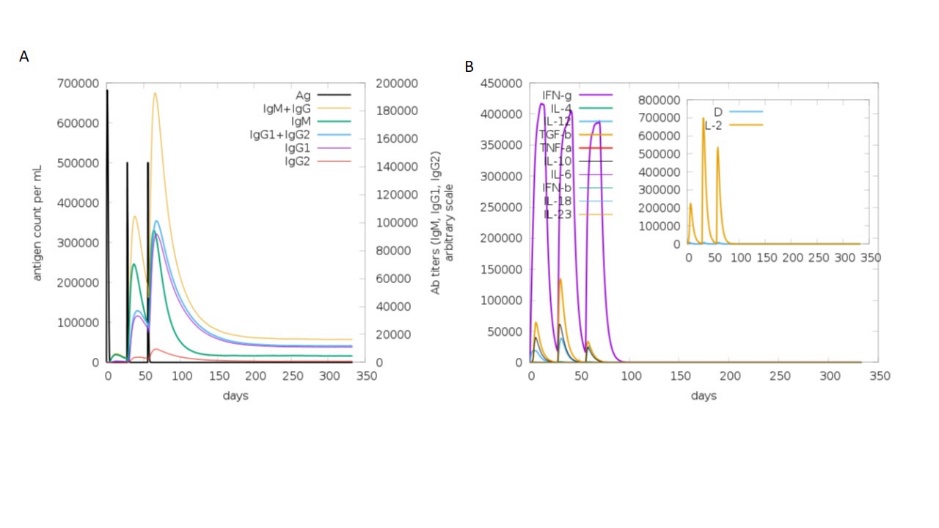


Supplementary Fig.5 : (A) B cell (B) PLB cell population, (C) B cell population per state, (D) TH cell population (E)TH cell population per state.


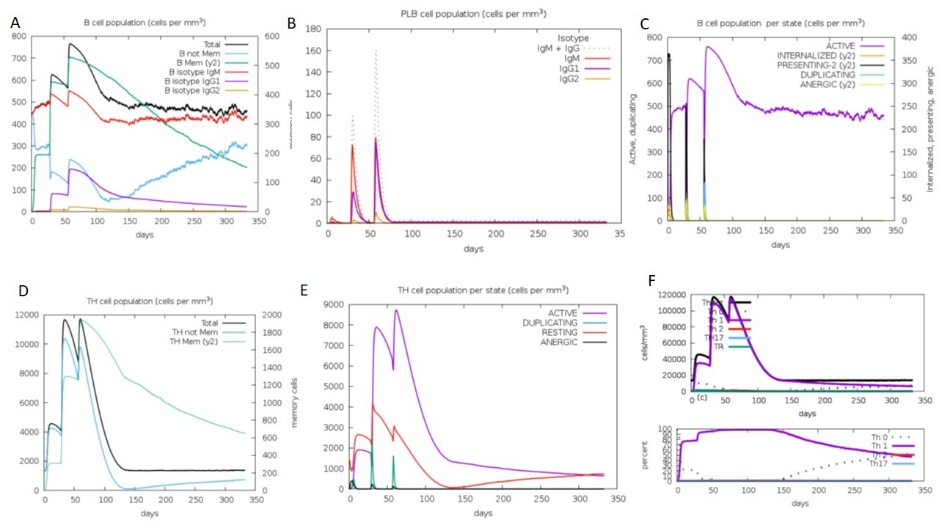


Supplementary Fig.6 : (A) TC cell population, (B)TC population per state, (C) NK cell population, (D) MA population per state, (E) DC population per state (F) EP population per state.


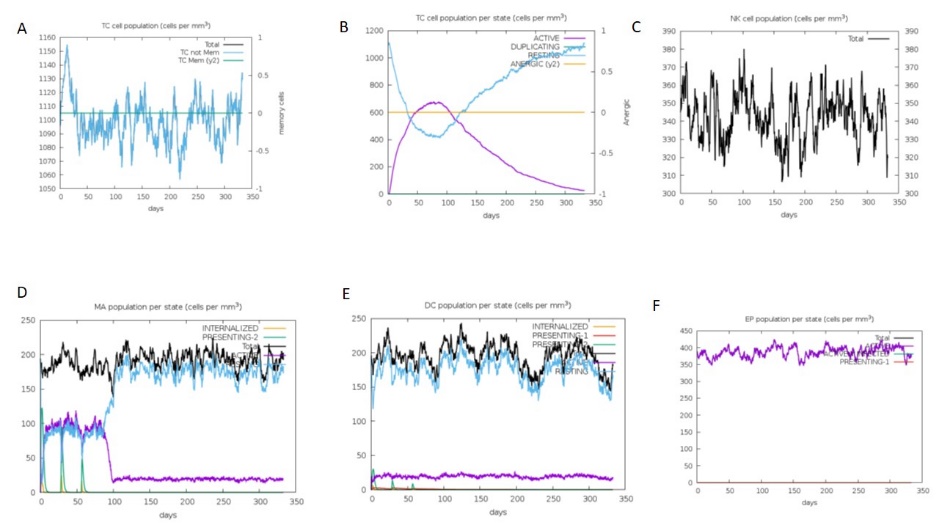

Supplement: Supplementary file 1 — Additional file 1: Table S1. IFN-γ Results for the selected B-cell, HTL and CTL epitopes of CABP proteins. Table S2. ToxinPred results for the selected B-cell, HTL and CTL epitopes of CABP proteins. Fig. S1. (A) Ramachandran plot showing the presence of amino acid residues in favoured, allowed and outlier region, (B) after refinement Ramachandran plot showing the presence of amino acid residues in favoured, allowed and outlier region. Fig. S2. ProSA predicted 3D structure showed Z-score-8.25. Fig. S3. The energy plot for all residues showed that most of the residues lie in the negative region. Vaccine Sequence Adjuvant Followed by Bcell epitopes, HTL epitopes and CTL epitopes and the linker. Fig. S4. (A) Antigen and immunoglobulins, (B) production of cytokine and interleukins. Fig. S5. (A) B cell, (B) PLB cell population, (C) B cell population per state, (D) TH cell population, (E) TH cell population per state. Fig. S6. (A) TC cell population, (B) TC population per state, (C) NK cell population, (D) MA population per state, (E) DC population per state, (F) EP population per state. [file 12865_2022_535_MOESM1_ESM.docx]
